# A model for de novo pigmentation of amelanotic retinal pigment epithelial cells

**DOI:** 10.1111/aos.17572

**Published:** 2025-09-02

**Authors:** Santosh Gupta, Lyubomyr Lytvynchuk, Taras Ardan, Hana Studenovska, Georgina Faura, Lars Eide, Ljubo Znaor, Slaven Erceg, Knut Stieger, Jan Motlik, Goran Petrovski

**Affiliations:** ^1^ Center for eye Research and Innovative Diagnostics, Department of Ophthalmology Institute for Clinical Medicine, Faculty of Medicine, University of Oslo Oslo Norway; ^2^ Department of Ophthalmology Justus Liebig University Giessen, University Hospital Giessen and Marburg GmbH Giessen Germany; ^3^ Karl Landsteiner Institute for Retinal Research and Imaging Vienna Austria; ^4^ Institute of Animal Physiology and Genetics Academy of Sciences of the Czech Republic Libechov Czech Republic; ^5^ Institute of Macromolecular Chemistry Academy of Sciences of the Czech Republic Prague Czech Republic; ^6^ Department of Medical Biochemistry Institute of Clinical Medicine, University of Oslo Oslo Norway; ^7^ Department of Ophthalmology University of Split School of Medicine and University Hospital of Split Split Croatia; ^8^ Research Center ‘Principe Felipe’, Stem Cell Therapies in Neurodegenerative Diseases Laboratory Valencia Spain; ^9^ Institute of Experimental Medicine, Academy of Sciences of the Czech Republic Prague Czech Republic; ^10^ Department of Ophthalmology Oslo University Hospital Oslo Norway

**Keywords:** in vitro model, L‐DOPA, melanosomes, pigmentation, RPE

## Abstract

**Purpose:**

Retinal Pigment Epithelial (RPE) cells perform critical functions in the visual cycle. Their melanin pigmentation, which is organized into specialized compartments – melanosomes, is highly critical for proper vision. A chemical method to induce pigmentation in a non‐pigmented model of ARPE‐19 cells was applied using L‐DOPA as a repurposed drug from the current treatment of Parkinson's disease.

**Methods:**

L‐DOPA was optimized for its toxic effect on ARPE‐19 cells along with pigmentation development. Gene expression and immunocytochemistry confirmed upregulation of melanogenesis‐related genes and proteins. Melanosomes were characterized by TEM.

**Results:**

We found 1000 μM L‐DOPA to induce pigmentation of ARPE‐19 cells by Day 3, and achieve full pigmentation by Day 5. By Day 5, L‐DOPA at 1000 μM induced mitochondrial and nuclear DNA damage. However, the gene expression of RPE‐specific markers (*tyrosinase*, *TYRP1*, *CRALBP*, *PEDF*) was significantly different in L‐DOPA‐treated ARPE‐19 cells compared to non‐treated ones. Positive expression for Tyrosinase enzyme was confirmed by ICC on both Day 3 and Day 5 of L‐DOPA treatment. Transmission electron microscopy showed the de novo melanosome formation with ultrastructural features of various stages of maturity (Stage I to IV), apical‐basal polarity and melanosome localization on the apical side of the L‐DOPA‐treated ARPE‐19 cells.

**Conclusion:**

Our study showed that L‐DOPA treatment could induce de novo melanosome formation in amelanotic RPEs. We propose a newer approach of developing an ex vivo model for de novo pigmentation of RPE cells with cell‐specific modification and culture condition optimization.

## 1 INTRODUCTION

The retinal Pigment Epithelial (RPE) cells, found in the posterior segment of the eye, between the choroid plexus and the neural retina, perform critical functions in the visual cycle. One of the characteristic features of the RPEs is their pigmentation due to melanin, which is organized in a specialized compartment known as melanosomes (Schraermeyer & Heimann, 1999; Yang et al., 2021). These melanosomes are synthesized during the early stage of fetal development, and eventually, melanogenesis stops after birth. Therefore, pigmentation remains unchanged throughout life without any turnover (Lopes et al., 2007; Schraermeyer et al., 2006). The RPE pigmentation plays an important role in attenuating light and its intensity that escapes the retina by filtering out reactive blue light, and therefore, it helps in enhancing the image formed by the retina (Booij et al., 2010). The melanin particles also absorb blue light and act as a natural scavenger of free radicals and protect the retina (Boulton & Dayhaw‐Barker, 2001) and choroid (Peters et al., 2006) from photo‐oxidative stress. Hence, pigmentation is highly critical for proper vision development and use. In certain disease conditions like albinism, the RPEs are non‐pigmented, which leads to progressive damage of the retina due to an absence of pigmentation in this cell layer and consequently affects vision. There exist no treatment modalities for inducing melanogenesis in RPEs in vitro, ex vivo or in vivo using conventional pharmacological strategies.

It has been reported that the pigmentation in human RPEs decreases with aging. It has also been shown that melanosomes in the RPEs from different regions of the eye significantly decrease by age of 40 (Feeney‐Burns et al., 1984; Weiter et al., 1986; Schmidt & Peisch, 1986). In another study, where comparisons were made between three age groups (1–20, 21–60 and 61–100 years), the decline in melanosomes in the macular RPEs between early and late decades was about 35%. This loss was correlated with an increase in the complex melanin granules (Feeney‐Burns et al., 1984). Similarly, it has been observed that primary RPEs lose pigmentation when cultured in vitro over the period of multiple passages (Fronk & Vargis, 2016). It could be inferred that the RPEs might not have an alternative mechanism to replenish lost melanosomes by de novo melanosome biogenesis or activation of the melanin synthesis pathway in humans.

An approach to artificially re‐pigment non‐pigmented ARPE‐19 cell model by reintroducing isolated melanosomes from porcine RPEs using a drug with melanin binding affinity has been reported (Hellinen et al., 2019). However, post‐pigmentation retainment of the melanosomes was not studied further. Another study showed that prolonged cultivation in pyruvate and DMEM/F12 resulted in re‐pigmentation of the ARPE‐19 cells (Ahmado et al., 2011). Furthermore, silk‐derived protein sericin can induce pigmentation in human RPEs (Eidet et al., 2016), but the melanosomes were not characterized extensively and the authors lacked showing widespread melanogenesis within a 14‐day treatment time. Interestingly, sericin has been shown to inhibit the enzyme Tyrosinase (Kato et al., 1998), which is the master enzyme associated with melanin synthesis and melanosome maturation.

L‐DOPA (L‐3,4‐dihydroxyphenylalanine) is a small molecule – a drug currently used in the treatment of Parkinson's (Molloy et al., 2005) and Alzheimer's disease (Martorana et al., 2008). L‐DOPA is also produced by melanin synthesizing cells like the melanocytes in the skin and the RPEs in the eye. The molecule is formed by the activity of the rate‐limiting enzyme tyrosinase from the precursor tyrosine, and it is subsequently used as a substrate for melanin polymer synthesis in the RPEs (Mishima, 1994). Studies in the late 1980s have explored the effect of tyrosine and L‐DOPA on inducing melanization in melanoma cell lines. It was shown that both molecules have a positive feedback regulation on the tyrosinase enzymatic activity, leading to increased melanization in this cell line (Slominski et al., 1988). Similarly, other studies have focused on the effect of L‐DOPA upon pheomelanogenesis in a mouse melanoma cell line and elucidating the enzyme kinetics on the catalysis of L‐DOPA (Sato et al., 1987). Although these studies have been performed on a melanoma cell line, the melanogenesis regulation is different in melanoma cells compared to RPEs – for the former, melanogenesis occurs throughout the lifetime while for the latter, it stops after birth. These studies highlight the role of L‐DOPA on melanosome biogenesis in vitro. With a focus on the retina, the effect of L‐DOPA on the developing retina has been shown to improve the retinal structure and visual function (Lee et al., 2019). L‐DOPA could be supplemented during the critical period of neuroplasticity in a mice model of human albinism. However, no studies have shown the effect L‐DOPA has on inducing de novo pigmentation in amelanotic RPE cells.

ARPE‐19 is a cell line derived spontaneously from human RPE cells. These are non‐pigmented, yet have features that limit their use as an alternative tool for studying replicative senescence, chromosomal abnormalities and immature phenotype in RPE cells (Pfeffer & Fliesler, 2022).

Our objective was to study the effect of L‐DOPA in inducing melanogenesis and pigmentation in ARPE‐19 cells and to develop a model system for pigment induction in a non‐pigmented RPE cell line in vitro. We studied the effect of L‐DOPA upon melanogenesis‐related genes and proteins expression in ARPE‐19 cells, as well as assessed the improvement in the cellular phenotype after treatment ultrastructurally.

## 2 MATERIALS AND METHODS

### 2.1 Materials

3‐(3,4‐Dihydroxyphenyl)‐l‐alanine, l‐3‐Hydroxytyrosine (L‐DOPA), Cat. No. – D9628, was purchased from Sigma‐Aldrich, USA. DMEM: F12 and Fetal Bovine Serum were purchased from Thermo Fisher Scientific, USA. All the other materials used were of cell culture grade.

### 2.2 Ethical clearance

Human primary RPE (hpRPE) isolation methodology was approved by the Regional Committees for Medical and Health Research Ethics for the Center for Eye Research and Innovative Diagnostics, Department of Ophthalmology, Oslo University Hospital and University of Oslo (REK No. 448416). All the procedures related to hpRPE experiments in this study were performed in accordance with the Guidelines of the Declaration of Helsinki.

### 2.3 Human primary RPE isolation and culture

Human primary RPEs (hpRPEs) from the rest of the material in the departmental Cornea Bank were isolated from human cadaver eyes and cultured using the protocol described in our previous publications (Lytvynchuk et al., 2022). Upon isolation, the hpRPEs were cultured in DMEM/F12 with 10% FBS without penicillin–streptomycin. The hpRPEs were then maintained in a humidified incubator at 37°C with an atmosphere of 5% CO_2_.

### 2.4 Cell culture

ARPE‐19 cells obtained from ATCC were used as a model for non‐pigmented RPE cells. The cells were cultured in DMEM/F12 (1:1 mixture of Dulbecco's modified Eagle's medium and Ham's F12) with heat‐inactivated 10% Fetal Bovine Serum (FBS), 1% Glutamax and without Penicillin–Streptomycin antibiotics. All cultures were maintained in a humidified incubator at 37°C with an atmosphere of 5% CO_2_ and 95% air. L‐DOPA was dissolved in cell culture‐grade water to prepare a 15 mM stock solution. Further dilutions (10 μM to 1000 μM) were prepared in complete media with FBS for cell treatment. Media were changed every single day until Day 5 of the study.

### 2.5 Viability assay

Presto blue (Cat. No A13261; Invitrogen, USA) assay was used in order to test the viability of ARPE‐19 cells after L‐DOPA treatment on Day 1, Day 3 and Day 5. After the treatment time, the medium was removed and the Presto blue reagent was added in a 1:10 ratio with complete DMEM/F12 with 10% FBS. After 75 min of incubation at 37°C, absorbance was measured immediately at 590 nm in a microplate reader (Victor3, 1420 Multilabel counter, Perkin Elmer, USA). Non‐treated ARPE‐19 cells were used as a control, while Presto blue: media (1:1) was used as a blank. *n* for the experiment was 4. MTT assay was performed using cell proliferation kit (Cat. No. 11 465 007 001), Roche, USA following the manufacturer protocol. Briefly, 7000 ARPE‐19 cells were seeded per well in a 96 well plate. Cells were treated with L‐DOPA (0 μM to 1000 μM) and spectrophotometric readings were recorded at Day 1, Day 3 and Day 5.

### 2.6 Melanin quantification

Melanin was quantified spectrophotometrically in L‐DOPA treated ARPE‐19 cells as described previously (Chung et al., 2019). Briefly, 2 × 10^5^ cells were collected in an Eppendorf tube for each group. 100 μL of 1 N NaOH containing 10% DMSO was added to the pellet and heated at 80°C for 90 min. Absorbance was then measured at 490 nm using a multimode plate reader (Vicotor3™, Perkin Elmer, USA). To convert the absorbance value to the amount of melanin, a standard curve was obtained from 0 to 500 μg/mL of synthetic melanin (Sigma‐Aldrich) solution dissolved in 1 N NaOH. The absorbance was averaged from three wells, and each experiment was performed in triplicates.

### 2.7 Quantitative RT‐PCR

Total RNA was isolated from the cell sample using a Qiagen microRNA extraction kit (Cat. No 74134, Qiagen, USA) following the manufacturer's protocol. RNA was quantified using Nanodrop. Reverse transcription of 1 μg of RNA from each sample was performed using the SuperScript III First Strand kit (Cat. No – 18080051, Invitrogen, USA). cDNA was amplified using the Power SYBR Green Mix (Cat. No – 43‐676‐59; Applied Biosystems, USA) using the ABI Prism7000 Sequence Detection System (Applied Biosystems). TaqMan probes for *tyrosinase*, tyrosinase related protein 1 (*TYRP1*), Mitf‐regulated pigment‐related genes (*MLANA*), cellular retinaldehyde‐binding protein (*CRALBP*) and pigment epithelium‐derived factor (*PEDF*) were used for gene expression studies. The cycle parameters consisted of an initial denature step of 95°C for 10 min followed by 40 cycles of 95°C for 15 s and 60°C for 30 s. Raw data was processed using the comparative CT method by the formula 2^−ΔΔCt^. Each amplification reaction was performed in triplicate using 20 ng of cDNA for each sample. 18S was used as an internal control and the gene expression was normalized to the control (non‐treated cells) for final gene expression analysis.

### 2.8 Immunocytochemistry (ICC)

Control‐ and L‐DOPA treated ARPE‐19 cells (Day 3 and Day 5) were fixed with 4% paraformaldehyde in PBS for 20 min. Post‐fixation, cells were permeabilized in 0.25% Triton X100 in PBS for 30 min and blocked in 10% donkey serum in TBST (0.1% tween20 in PBS) for 45 min. After blocking, cells were incubated with primary antibody (Occludin, Tyrosinase‐cat no) overnight at 4°C, followed by secondary antibody (Alexa Flour 488 donkey, anti‐mouse, Alexa Flour 555 donkey, anti‐rabbit) incubation. The cells were counterstained and mounted using DAPI mounting media (Fluoroshield™ with DAPI, Merck, USA).

### 2.9 Mitochondrial and nuclear DNA damage and mitochondrial DNA copy number analyses

ARPE‐19 cells (non‐treated or treated for 3 and 5 days with L‐DOPA) were pelleted down and snap‐frozen for further use. DNA damage was determined from the ability to inhibit TaqI restriction digestion (was determined using a method described previously (Wang et al., 2016) with modification). DNA was extracted from the cells using the DNeasy blood and tissue DNA isolation kit (Cat. No ‐69504, Qiagen, USA). Briefly, cells were digested and centrifuged at 1000 *g* before ethanol precipitation to remove melanin. The following steps were followed as per the instructions provided by the manufacturer. A qPCR reaction mixture is prepared with or without the TaqI restriction enzyme. 12S and NDUFA9 primers, specific for mtDNA and nDNA, respectively, were used (Sequence: 12S – FP 5′‐AAA CTG CTG CTC GCC AGA‐3′, RP 5′‐CAT GGG CTA CAC CTT GAC CT‐3′; NDUFA9 – FP 5′‐GCA AGG GTC CCT ATG AGA GAA‐3′, RP 5′‐CAA GAA CGA GGG GAA AAG TG‐3′). The PCR program was run on a StepOne™ Real‐Time PCR System (Applied Biosystems™). mtDNA copy number (mtDNA‐CN) was calculated as a ratio between 12S and NDUFA9 copies (Longchamps et al., 2020).

### 2.10 Transmission electron microscope (TEM)

Control and L‐DOPA treated ARPE‐19 cells (Day 3 and Day 5) were fixed for 1 h at room temperature in TEM grade 2.5% glutaraldehyde in 0.1 M sodium cacodylate buffer (pH 7.4). The specimens were postfixed with 1% OsO_4_ and 1.5% potassium ferrocyanide in 0.1 M sodium cacodylate buffer at room temperature for 3 h, block‐stained with uranyl acetate overnight, dehydrated in a graded series of ethanol and embedded in epoxy resin. Ultrathin sections were post‐stained with lead citrate and examined under a transmission electron microscope (EM 902A, Zeiss).

### 2.11 Statistical analyses

All the in vitro experiments were performed in triplicates for statistical validation, and reproducibility was assessed by repeating each experiment at least three times. D'Agostino‐Pearson test for normality was performed before statistical analysis among various groups using one‐ or two‐way analysis of variance (ANOVA) followed by Tukey's post hoc test using GraphPad Prism software (Version 9, USA) unless otherwise mentioned. Differences were considered statistically significant at *p < 0.05, **p < 0.1, ***p < 0.001. Data are presented as the mean of three independent experiments as mentioned in each figure panel unless otherwise noted.

## 3 RESULTS

### 3.1 Viability of ARPE‐19 upon L‐DOPA treatment

**FIGURE 1:**
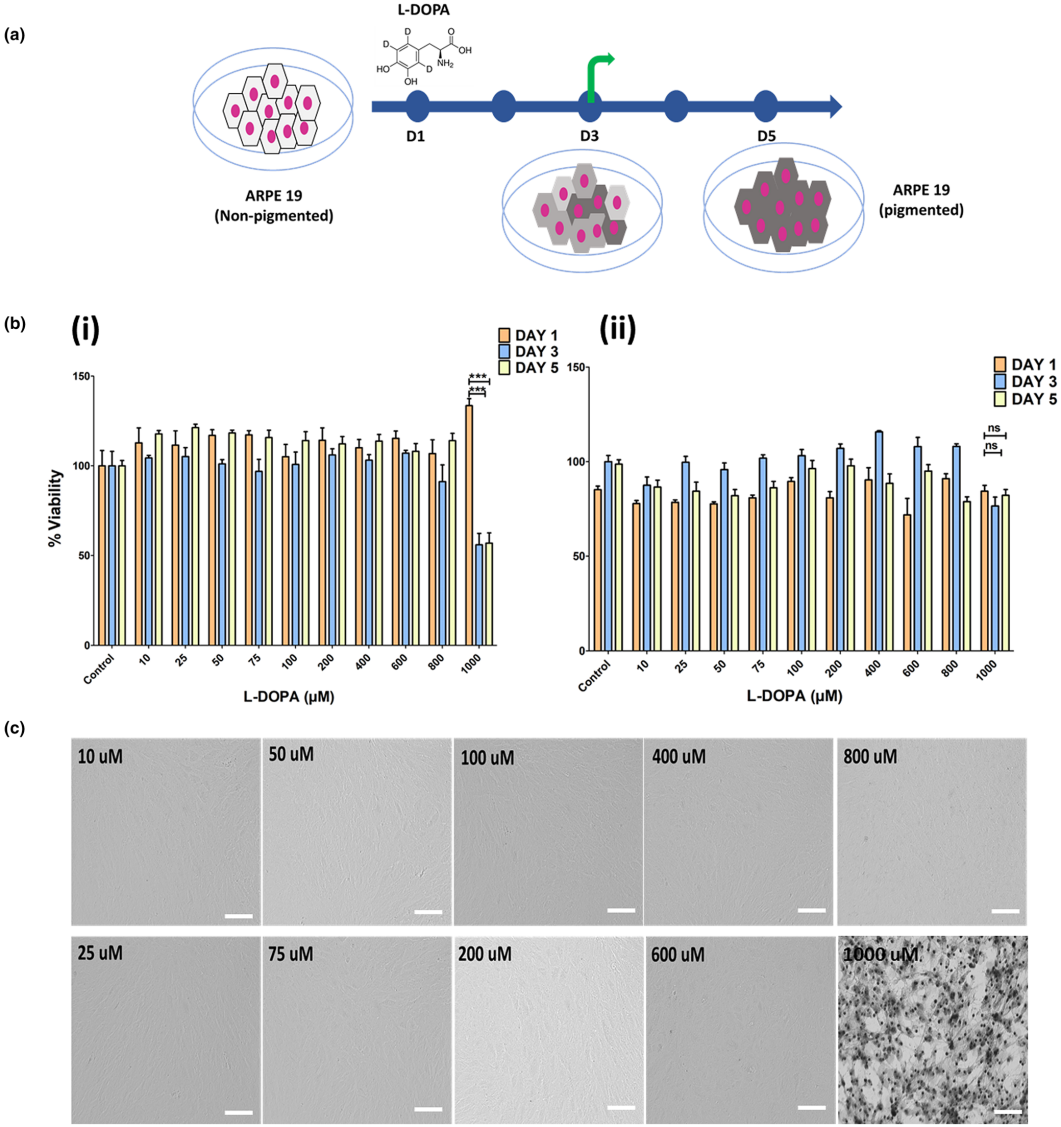
Viability of ARPE‐19 upon L‐DOPA treatment. (a) Presentation of L‐DOPA administration. (b) (i) Presto blue viability assay – viability of cells exposed to increasing concentration of L‐DOPA for 1, 3 and 5 days. 1000 μM L‐DOPA resulted in a decrease in viability, irrespective of whether exposure was 3 or 5 days. No significant difference could be found between Day 3 and Day 5 groups. (ii) MTT viability test – 1000 μM L‐DOPA showed no significant toxicity at Day 5 compared to Day 3 and Day 1. (c) Brightfield microscopic images of the Day 5 group of L‐DOPA (10 μM to 1000 μM) treated ARPE‐19 are shown. Only the 1000 μM concentration could show visible pigmentation compared to other L‐DOPA treatment concentrations (10 to 800 μM). Scale bar: 50 μm. Data were analysed by a one‐way ANOVA method. The differences in cell viability between the groups were considered statistically significant when *p* < 0.05, and the *p*‐values shown are ***p < 0.001. Data are presented as the mean ± SD of three independent experiments.

L‐DOPA is an intermediate product formed during melanin synthesis. We wanted to check if L‐DOPA influences cell growth and causes any cytotoxicity in a dose dependent manner. Figure 1a outlines the study plan to assess the cytotoxicity and pigmentation induced by L‐DOPA on ARPE‐19 cells. The cytotoxicity by Presto Blue assay of L‐DOPA on ARPE‐19 was tested over a 5‐day period of treatment. L‐DOPA with a concentration range from 0 to 800 μM could not induce any significant cell death, whereas 1000 μM showed a significantly higher cell death after 3 and 5 days of treatment (Figure 1b(i)). MTT‐based cytotoxicity study showed no significant differences between day 3 and day 5 in viability (Figure 1b(ii)). Brightfield microscopic images of ARPE‐19 cells treated with various concentrations (10 to 1000 μM) of L‐DOPA for 5 days are shown in Figure 1c. Cells treated with 1000 μM for 5 days showed visible pigmentation, while no pigmentation could be observed for concentrations of L‐DOPA up to 800 μM.

### 3.2 L‐DOPA induces pigmentation in amelanotic ARPE‐19 cells

**FIGURE 2:**
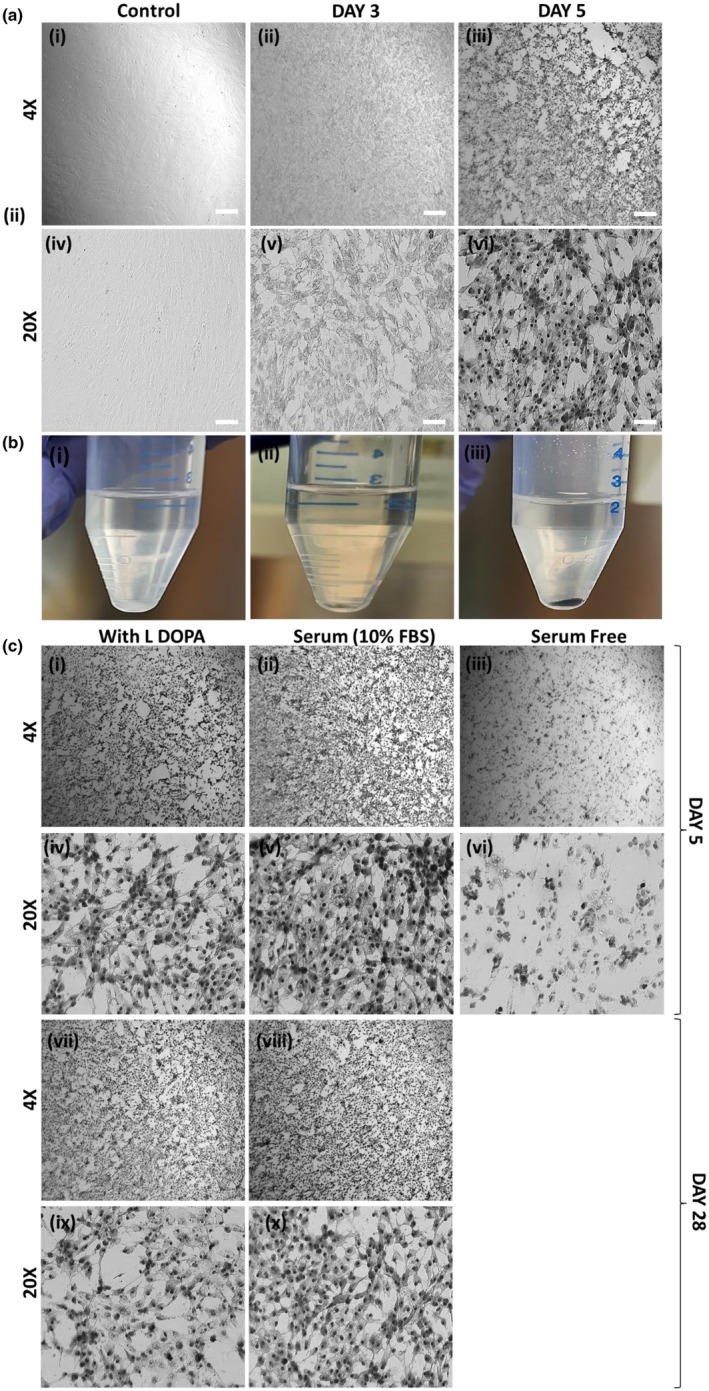
Morphological appearance of the ARPE‐19 cells treated by L‐DOPA. (a) Brightfield microscopic images of Control (non‐treated), L‐DOPA (1000 μM) treated ARPE‐19 cells on Day 3 and Day 5. The images were acquired at magnification: 4× (A (i), (ii) and (ii)) and 20× (A (iv), (v) and (vi)). Day 3 cells showed the appearance of pigmentation, while Day 5 cells showed improved pigmentation compared to the Control and Day 3 cells. (b) Photographic images of the cell pellet of (i) non‐treated ARPE‐19 cells; (ii) Day 3 and (iii) Day 5 L‐DOPA treated (1000 μM) ARPE‐19 cells. (c) L‐DOPA withdrawal results on 5 days and 28 days after a 5 days L‐DOPA/pigmentation treatment. (i) 4×, (iv) 20× magnified images of ARPE‐19 pigmented cells' morphology and proliferation, as well as state of pigmentation. (ii) 4× (v) 20× magnified image of the cells in the absence of L‐DOPA treatment showing improved proliferation and retention of pigmentation at Day 5 post‐pigmentation treatment. (iii) 4×, (vi) 20× magnified image of ARPE‐19 cells showing appearance of cell death and loss of pigmentation when cultured in the absence of L‐DOPA and FBS. (viii) 4× (viii) 20× magnified image of the cells in the presence of L‐DOPA and (ix) 4× and (x) 20× without L‐DOPA after 28 days post 5‐days pigmentation treatment. Scale bar: At 4× magnification – 100 μm, at 20× magnification – 50 μm.

L‐DOPA being an important metabolite that is used for melanin production, we wanted to see if exogenously added L‐DOPA could induce melanin synthesis in a non‐pigmented RP{E} cell model. To assess this, we cultured ARPE19 cells with different concentrations of L‐DOPA to see the induction of pentation. ARPE‐19 cells were treated with L‐DOPA (10 to 1000 μM) for 5 days and its effect upon pigmentation was assessed on Day 1, Day 3 and Day 5. Figure 2(I)(a) showed pigmentation induction by Day 3, which further improved by Day 5. Furthermore, the morphology of the L‐DOPA‐treated cells showed improved hexagonality at the 1000 μM concentration compared to the non‐treated ARPE‐19 in the present study. Figure 2(I)(b) shows the gross appearance of L‐DOPA‐treated ARPE‐19 cells upon centrifugation. The pigmentation‐induced ARPE‐19 showed black pellets both at Day 3 and Day 5 compared to non‐pigmented cell pellets found in the controls. We further tested the toxicity of L‐DOPA on ARPE‐19 cells as cell death in the pigmented group could be observed by the 1000 μM L‐DOPA treatment. To test the ability of the cells to retain pigmentation, L‐DOPA was removed from the media, and the cells were cultured in standard media (DMEM/F12, 10% FBS) (Figure 2c). Post‐pigmentation (Day 5) L‐DOPA‐treated cells after 4 days of absence of L‐DOPA treatment continued growing and maintained good RPE cell morphology (Figure 2c(ii,v)), whereas the groups with continuous L‐DOPA treatment could show less growth (Figure 2c(i,iv)). The 5‐day pre‐pigmented ARPE‐19 cells when cultured in serum‐free conditions and without L‐DOPA appeared to undergo cell death (Figure 2c(iii,vi)).

**FIGURE 3:**
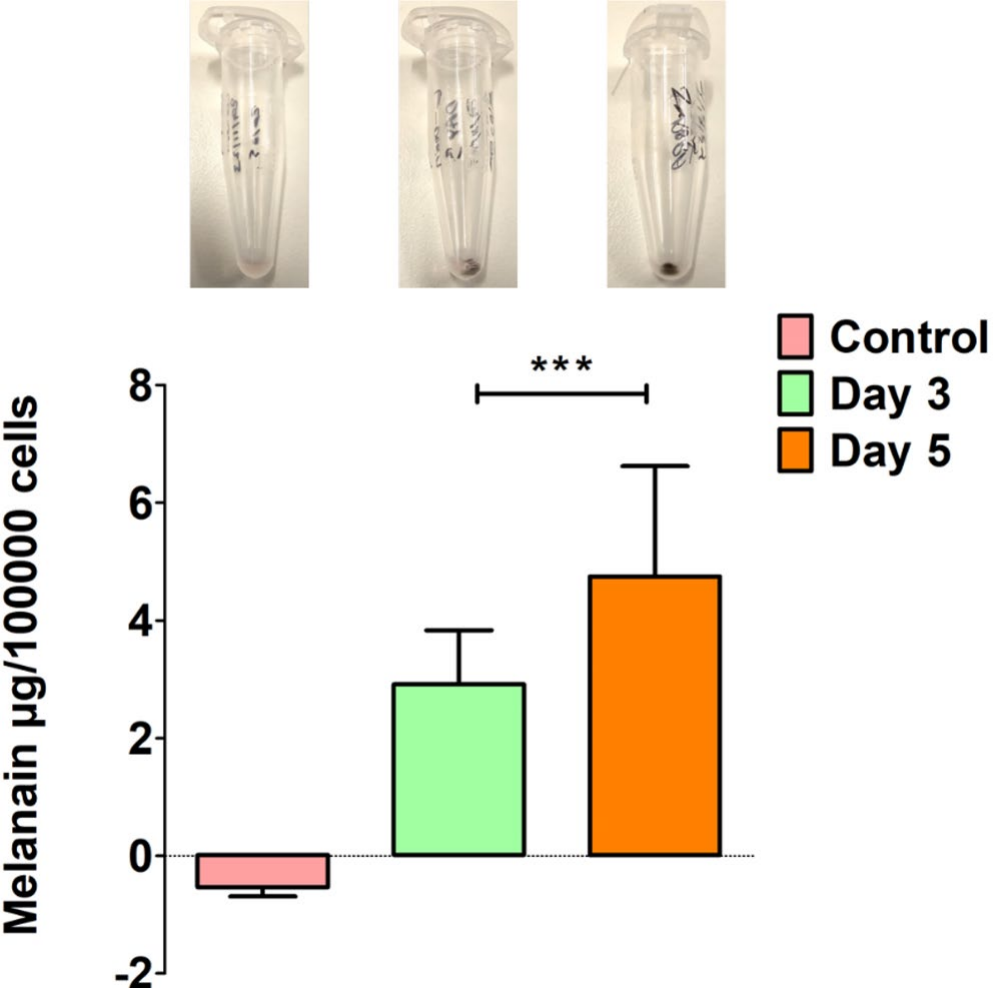
Melanin quantification in L‐DOPA induced pigmented ARPE‐19 cells. Melanin was quantified in control cells (−0.534 μg), Day 3 (2.919 μg), Day 5 (4.752 μg) per 100 000 cells. Photographic images of the cell pellet in the Eppendorf represent control (non‐treated), Day 3 and Day 5 L‐DOPA treated cells. Data are presented as the mean ± SD of three independent experiments. ***p < 0.001.

Melanin was quantified in the control ARPE‐19 cells (0.534 μg), and Day 3 (2.919 μg) and Day 5 (4.752 μg) L‐DOPA treated cells accordingly (Figure 3). Melanin was significantly higher in Day 5 vs. Day 3 L‐DOPA treated group.

### 3.3 Mitochondrial and nuclear DNA damage in response to L‐DOPA treatment

**FIGURE 4:**
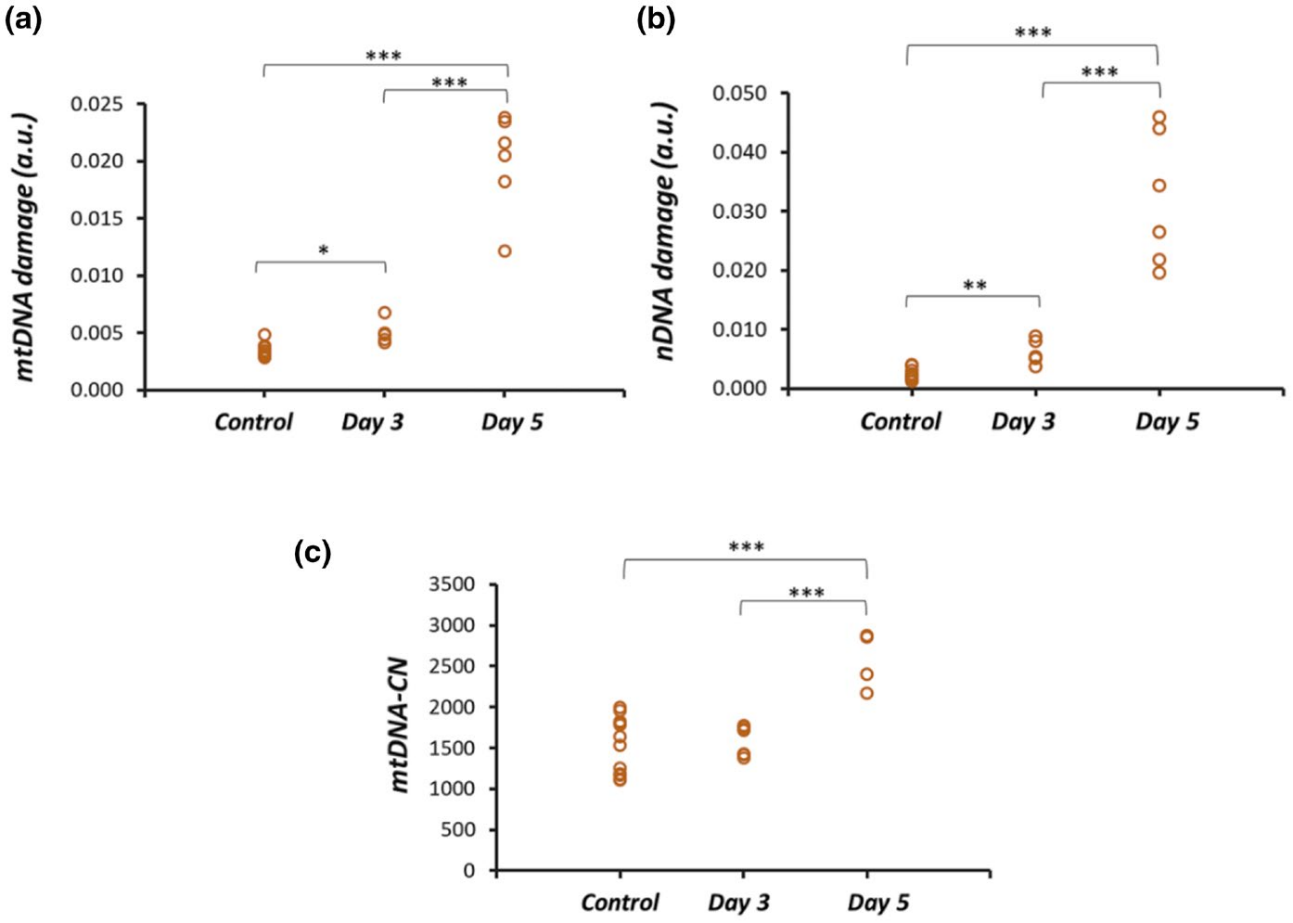
Effect of L‐DOPA (1000 μM) treatment on (a) mitochondrial and (b) nuclear DNA and mitochondrial copy number. (c) Mitochondrial DNA damage was significantly higher in the Day 5 group (*p* > 0.001) compared to the Control and Day 3 groups. Data are presented as the mean ± SD of three independent experiments. *p < 0.05, **p < 0.01, ***p < 0.001.

L‐DOPA is formed due to enzyme‐mediated oxidative process. The addition of L‐DOPA could lead to ROS formation, and to assess the effect of exogenously added L‐DOPA, we studied the mitochondrial and nuclear DNA damage in the pigmented ARPE19 cell. L‐DOPA treatment induced mitochondrial DNA (mtDNA) damage both in Day 3 and Day 5 treated ARPE‐19 cells compared to Control. The mtDNA damage was significantly lower between Control and Day 3 groups vs. Control and Day 5 (Figure 4a). A similar trend was also observed in the case of nuclear DNA (nDNA) damage, where such damage was significantly higher on Day 5 compared to Day 3 vs. Control cells (Figure 4b). There was no difference in the copy number variations between the Control and Day 3 groups. However, there was a significant increase in mitochondrial copy number in the Day 5 group compared to the Day 3 group (Figure 4c).

### 3.4 Enhancement of mature RPE‐specific gene‐ and protein‐ expression upon L‐DOPA treatment

**FIGURE 5:**
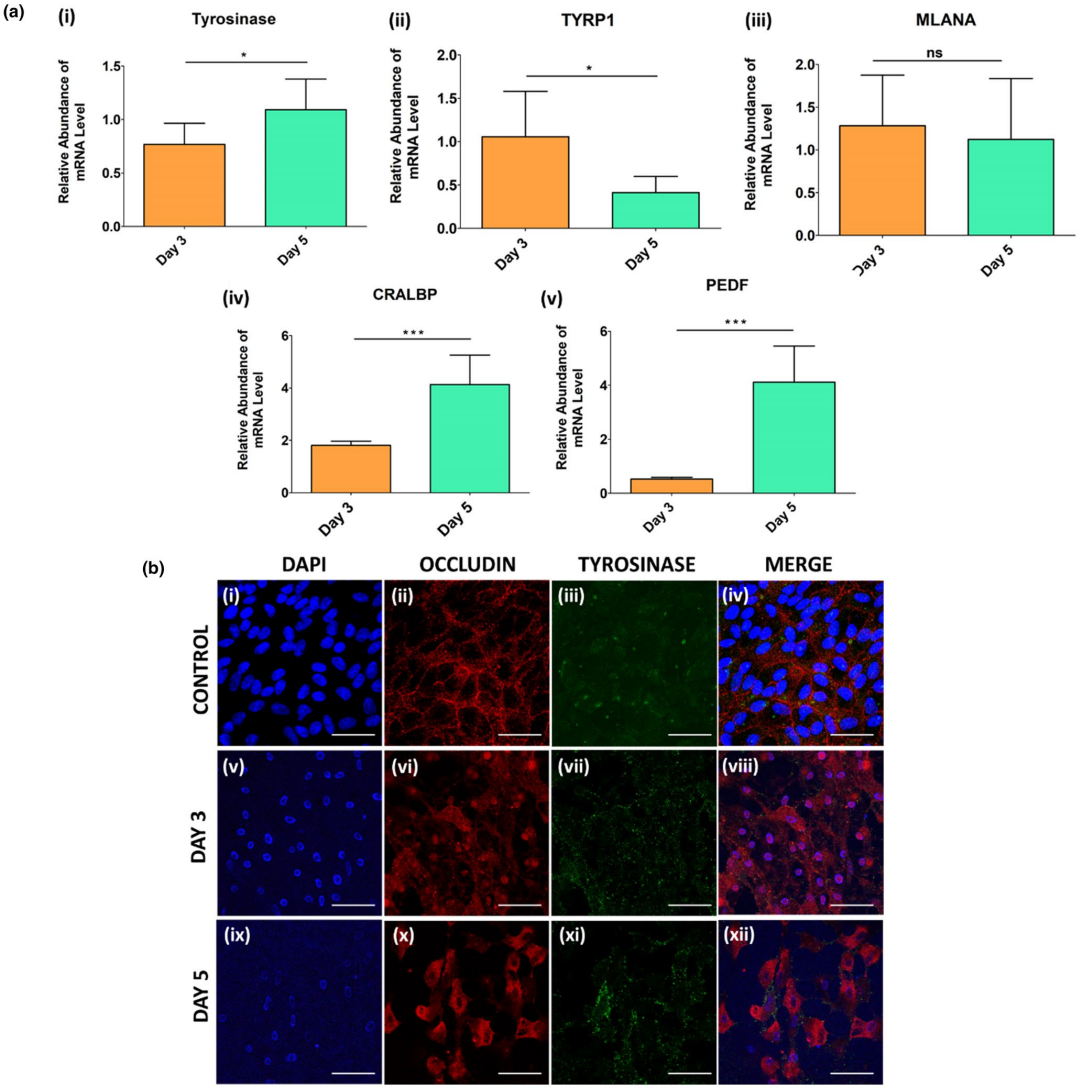
Gene‐ and protein expression of melanogenesis and RPE markers. (a) Quantitative RT‐PCR of RPE‐specific gene expression after L‐DOPA (1000 μM) treatment. (i) *Tyrosinase*, (iv) *CRALBP* and (v) *PEDF* gene expression were significantly higher in the Day 5 group compared to the Day 3 group. (ii) *TYRP1* was significantly higher in the Day 3 group vs. Day 5 group. (iii) *MLANA* showed no significant difference between Day 3 and Day 5. (b) Immunocytochemistry showed RPE‐specific Tyrosinase and Occludin protein expression in Control (i), Day 3 (ii) and Day 5 L‐DOPA treated groups. The Day 5 group showed higher expression of Tyrosinase compared to the Control and Day 3 groups. Occludin protein expression was observed in all three groups (Control, Day 3 and Day 5). The gene expression data for Day 3 and Day 5 presented here were normalized to the Control (non‐treated) values. A one‐way ANOVA test was performed for statistical evaluation. Differences were considered statistically significant at *p < 0.05, ***p < 0.001. Data are presented as the mean ± SD of three independent experiments. Scale bar: 50 μm.

Induction of pigmentation signifies activation of the melanogenesis pathway and other mature RPE‐specific genes. Since we observed induction of pigmentation, we further studied the effect of L‐DOPA on mature RPE‐specific gene and protein induction. qPCR and ICC studies were performed to assess the changes in RPE‐specific gene expression, with emphasis on the enzyme Tyrosinase. The results showed significantly higher gene expression of the *tyrosinase* enzyme in Day 5 compared to the Day 3 group (Figure 5(i)). Other genes associated with melanin synthesis, like *TYRP1*, were also upregulated at Day 3, and significantly lower by Day 5 (Figure 5(ii)); *MLANA* showed no significant differences between Day 3 and Day 5 of L‐DOPA treatment (Figure 5(iii)). Genes related to a functional property of the RPEs, such as *CRALBP* (Figure 5(iv)) and *PEDF* (Figure 5a(v)) were found to be significantly higher in the Day 5 compared to Day 3 group. Similarly, the protein expression of Occludin (Figure 5b(i)) and Tyrosinase (Figure 5(ii)) was found in both Day 3 and Day 5 groups by ICC. Tyrosinase protein expression appeared punctuated, and it was distributed throughout the cytoplasm and the nuclear membrane (Figure 5b(xi)).

### 3.5 L‐DOPA treatment induces cytoplasmic melanin‐like particles formation detected by transmission electron microscopy

**FIGURE 6:**
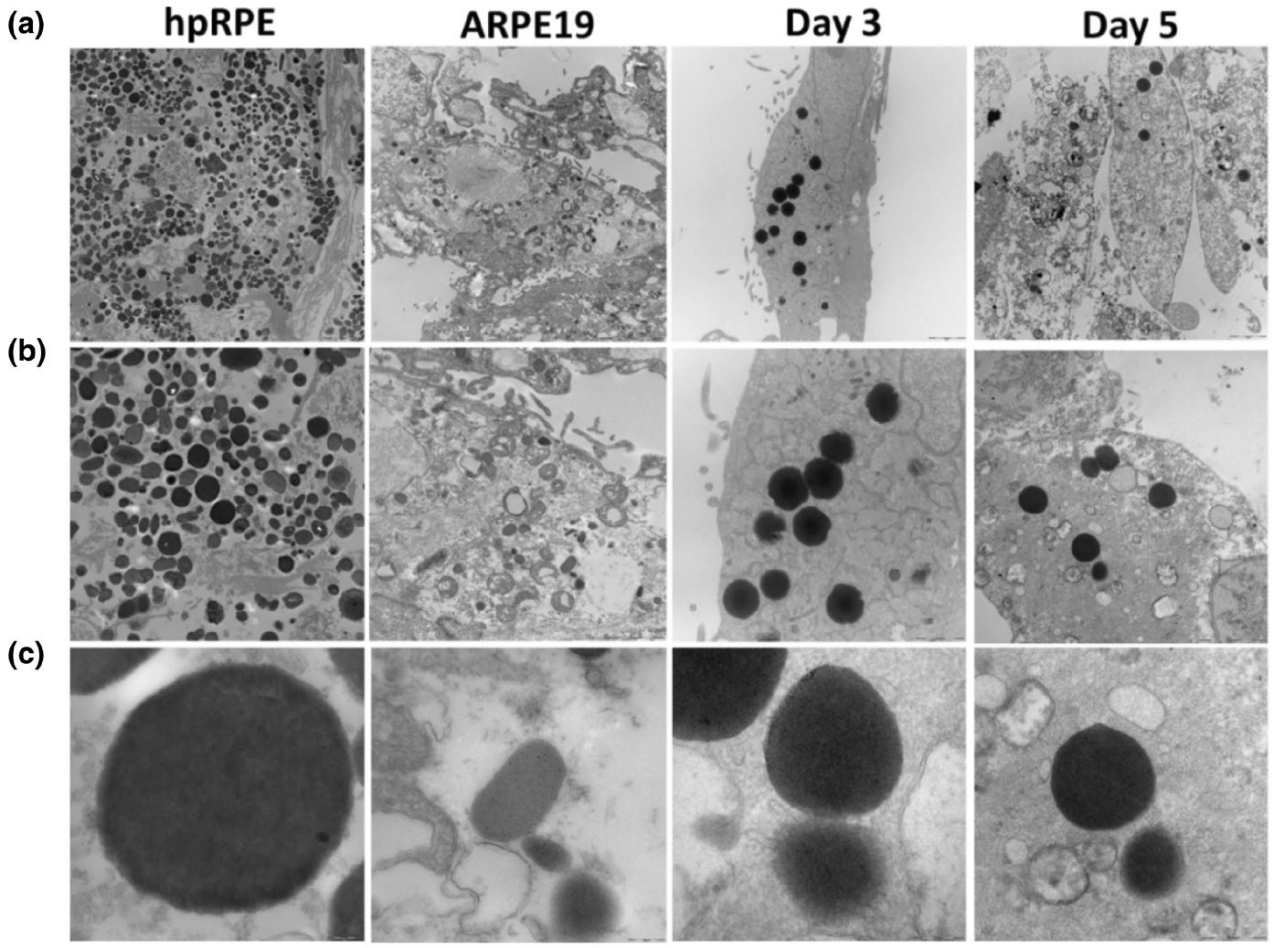
Transmission electron microscopic analysis of the pigmentation of the human primary RPEs (hpRPEs) and the ARPE‐19 cells upon L‐DOPA (1000 μM) treatment after Day 3 and Day 5. (a) Images of the hpRPE, ARPE‐19 and Day 5 L‐DOPA treated cells showing the presence of melanosomes. Day 3 had a negligible presence of melanosomes. (b) images showing a higher magnification of the apical side of the cell and the distribution of the melanosomes in hpRPE, Day 3 and Day 5 L‐DOPA treated ARPE‐19 cells. Control ARPE‐19 cells had no melanosomes. (c) Higher magnification image showing mature (Stage IV) melanosomes in hpRPE, Day 3 and Day 5 L‐DOPA treated ARPE‐19 cells.

Since we observed pigmentation induction, we wanted to study the ultrastructure of melanosomes and compared them to primary RPE cells to find out similarities between naturally formed melanosomes and artificially induced melanosomes in a non‐pigmented RPE cell model. The hpRPE showed a typical high abundance of melanosomes distributed in the cytoplasm. Cells were observed in the Day 3 and Day 5 groups after L‐DOPA treatment of ARPe‐19 cells, which without L‐DOPA treatment showed negligible to very low levels of melanosomes (Figure 6a). Melanosomes were distributed on the apical side of the cells for the hpRPE, as well as Day 3 and Day 5 L‐DOPA treated ARPE‐19 cells (Figure 6b). The absence of melanosomes in the non‐treated ARPE‐19 confirmed their amelanotic appearance. Figure 6c shows mature melanosomes (Stage IV) observed in a typical hpRPE. Similarly, mature melanosomes could be observed in Day 3 and Day 5 L‐DOPA treated ARPE‐19 cells.

### 3.6 L‐DOPA treatment maintains RPE‐like cellular phenotype

**FIGURE 7:**
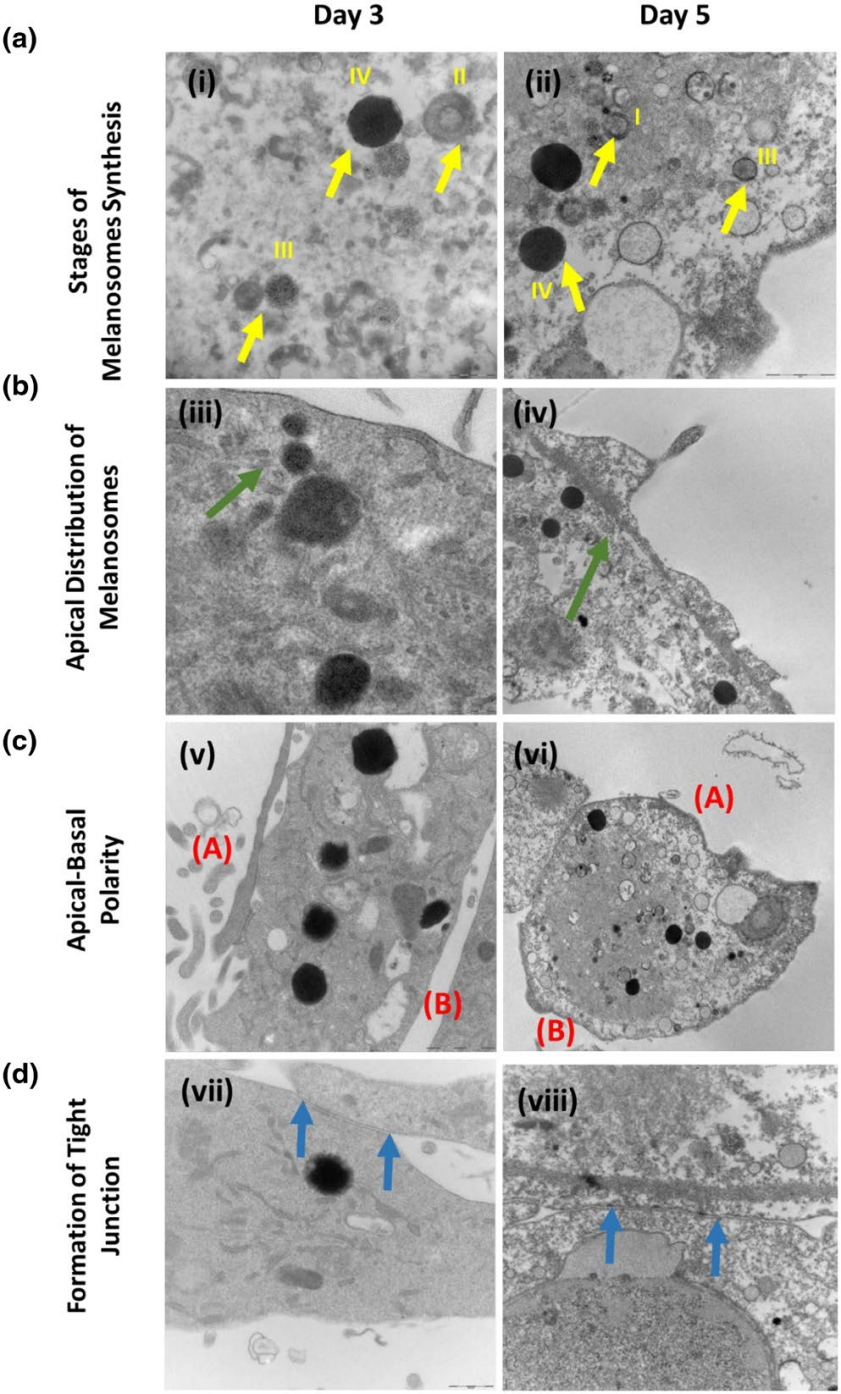
Ultrastructural features and cellular phenotype of the APRE‐19 cells treated by L‐DOPA. (a) Stages of the melanosomes could be observed in the ARPE‐19 cells after Day 3 and Day 5 of L‐DOPA treatment (Stage I to IV). (b) The apical distribution of the de novo melanosome formation could be observed in otherwise non‐pigmented ARPE‐19 cells after L‐DOPA treatment (the yellow arrow shows the presence of microvilli on the ARPE‐19 cells). (c) The apico‐basal polarity of L‐DOPA treated ARPE‐19 cells could be observed (A – Apical; B – Basal; red colour). (d) Formation of tight junctions could also be observed on both Day 3 and Day 5 of L‐DOPA treatment of ARPE‐19 cells (blue arrow).

We further wanted to confirm the ultrastructure features of artificially induced melanosomes by L‐DOPA by studying the properties of melanosomes and see if they resembled melanosomes of primary human RPE cells. The ultrastructural features of the ARPE‐19 cells treated by L‐DOPA were studied by transmission electron microscopy. Four features were explored to see the changes in ARPE‐19 cells upon L‐DOPA exposure: (1) Stage of melanosome formation, (2) Apicobasal polarity, (3) Apical distribution of melanosomes and (4) Formation of tight junctions between the cells. L‐DOPA (1000 μM) treatment induced melanosome formation as observed by the abundance of the various stages of melanosomes formed, indicating initiation of the melanosome‐formation pathway. Day 3 (Figure 7a(i)) and Day 5 ARPE‐19 cells showed the presence of (Stage I to IV) melanosome structures (Figure 7a(ii)). The melanosome distribution in the apical side of the ARPE‐19 cells on Day 3 (Figure 7b(iii)) and Day 5 (Figure 7b(iv)) could also be observed. The apical side of the cells is indicated in green arrows, showing the presence of cilial projections on the ARPE‐19 cells. The Apico‐basal polarity of the ARPE‐19 cells could be observed at both Day 3 (Figure 7c(v)) and Day 5 (Figure 7c(vi)), indicating maintenance of the epithelial state of these cells. Formation and maintenance of tight junctions is a functionally essential feature of the ARPE‐19 cell, and such junctions were observed at both Day 3 (Figure 7d(vii)) and Day 5 (Figure 7d(viii)) following L‐DOPA treatment. The tight junctions occurring between two cells are indicated by the blue arrows.

## 4 DISCUSSION

The availability of a model system to study melanogenesis in non‐pigmented RPE cells is limited due to the progressive loss of functionality and epithelial character in primary RPEs isolated from human eyes. This limits the usage of therapy development and high‐throughput drug screening for studying pigmentation‐inducing drug candidates or other therapeutic interventions for inducing pigmentation and melanogenesis in RPE cells. We hereby use a commonly applied cell line model of RPE cells – the ARPE‐19 cells, which exhibit various biomarker features of human RPEs with few exceptions, including lack of pigmentation (Pfeffer & Fliesler, 2022). In our study, we therefore used ARPE‐19 cells to better understand the effect of L‐DOPA and explore its ability to induce melanogenesis/pigmentation in an in vitro culture model.

L‐DOPA and Tyrosine both act on the rate‐limiting master enzyme Tyrosinase in the melanogenesis pathway (Boissy et al., 2006) (Körner & Pawelek, 1982). L‐DOPA is formed by oxidation of Tyrosinase, and it is further catalysed to form a subsequent substrate for melanin synthesis in the subcellular structures called melanosomes (Mishima, 1994). Therefore, we tested the effect of L‐DOPA on the viability and ability to induce pigmentation in ARPE‐19 cell lines. We observed significant cell death after exposing ARPE‐19 cells to L‐DOPA at a concentration of 1000 μM after 5 days of treatment by the Presto blue viability assay, whereas the MTT assay found no significant difference in the cell death between Day 3 and Day 5 of L‐DOPA treatment.

It has been reported extensively that L‐DOPA undergoes autoxidation, leading to the formation of reactive oxygen species (Kostrzewa et al., 2002). This is attributed to the cytotoxic effect of L‐DOPA observed in neuronal cells in vitro (Lai & Yu, 1997; Mena et al., 1992). The autoxidation is related to the oxygen tension, and cells cultured with as high as 1000 μM concentration of L‐DOPA could contribute to the observed cytotoxic effect. The cell death could also be attributed to the reactive oxygen species being formed by the auto‐oxidation of L‐DOPA in the media. This can be further substantiated by the observation of increased mtDNA and nDNA damage on Day 5 compared to Day 3 and Control untreated ARPE‐19 cells.

Melanin presence in the RPEs is a mixed polymer synthesized from α‐tyrosine in a specialized subcellular compartment called melanosomes (Borovanský & Riley, 2011). L‐tyrosine and L‐DOPA are in fact consecutive substrates for the same multifunctional enzyme, Tyrosinase (Körner & Pawelek, 1982). It has been shown by Slominski et al. (1988) that Tyrosine and L‐DOPA act through related, yet distinct, mechanisms to increase melanin production in a melanoma cell line by increasing the melanosome formation and tyrosinase expression. This correlates well with our findings that L‐DOPA could increase the *tyrosinase* expression level temporally. Upregulation of *Tyrosinase* and *TYRP1* genes is associated with melanin synthesis, and the *MLANA* gene is associated with melanosome formation (Gibbs et al., 2004). This indicates that both melanin synthesis and melanosome formation/assembly were activated upon L‐DOPA treatment leading to pigmentation.

In our study, we found that L‐DOPA treatment could enhance the gene expression for *CRALBP* and *PEDF*, both genes being critical for the vision cycle and angiogenesis regulation, respectively, exhibited by the RPEs. We also found improvement in the hexagonality of the L‐DOPA‐treated ARPE‐19 cells. All these factors underline an improvement in the functional features of the ARPE‐19 cells treated by L‐DOPA.

Ultrastructural characterization of the L‐DOPA treated ARPE‐19 cells showed many features indicating activation of melanosome formation and improved cellular phenotypes. Melanogenesis can be categorized into four stages starting from Stage I, where a vesicular structure destined for melanin deposition is formed (Boissy et al., 2006). We observed many such structures in the L‐DOPA treated cells. Stage II pre‐melanosomes show striations on which melanin can be deposited (McGlinchey et al., 2011) (Boissy et al., 2006). In Stage III melanosomes, the structures contain both visible striations and melanin deposition (Boissy et al., 2006). We observed similar structures as found in Stages II and III in both Day 3 and Day 5 groups following L‐DOPA treatment. Stage II pre‐melanosomes with striations could be seen as early as Day 3 treated cells, and Stage III structures containing striations and melanin deposition could be observed at Day 5. Finally, Stage IV shows fully mature melanosomes with complete melanization and striations not visible (Boissy et al., 2006). Although Stage IV structures were not so abundant as observed in the hp. RPEs, they could be observed in the L‐DOPA treated ARPE‐19 cells with identical morphology, which confirms activation of the melanosome machinery upon L‐DOPA treatment. It is important to mention here that a major part of melanosome biogenesis takes place during in utero development, while melanin deposition takes place until the age of 2 years (Stroeva & Mitashov, 1983). Although it has been reported that classical pre‐melanosomes essential for pre‐natal melanogenesis are not essential for melanogenesis in adult RPE cells (Biesemeier et al., 2010), in our study, we could detect the characteristic stages of melanosome biogenesis including pre‐melanosomes (Stage I and II) in the L‐DOPA treated ARPE‐19 cells. This requires further exploration about how non‐classical melanosome biogenesis pathways are activated in terminally differentiated RPE cells. Our study, therefore, provides for the first time, experimental evidence that a conventional small molecule can be used to induce melanogenesis in a cell model that is rendered by an inactive melanosome biogenesis machinery.

The distribution of the melanosomes within the RPEs has been shown to play an important role in the photoprotective function (Różanowski et al., 2008), and therefore, highlights one important feature that could exist in a model system representing RPEs of the human eye. Our observation aligns with the previously published data that the spherically shaped melanosomes are distributed towards the apical side of the RPE cells, along the line of the apical projections with size‐dependent distribution from the apical towards the basal side (Pollreisz et al., 2018; Pollreisz et al., 2020).

RPEs exist as a monolayer of epithelial cells with a prominent apical–basal polarity (Lehmann et al., 2014; Rizzolo, 2007) and microvilli on the apical side (Peng et al., 2003). In our study, L‐DOPA‐treated ARPE‐19 cells exhibited apical microvilli structures. The apicobasal polarity also regulates the secretion of growth factors on the respective side, such as VEGF and PEDF – (Sonoda et al., 2009). Interestingly, in our study, we found that L‐DOPA treatment could enhance the gene expression of *PEDF*, an inference indicating the improved apicobasal polarity of the treated ARPE‐19 cells. Our observation also correlates with another study where a lower concentration of L‐DOPA could increase PEDF secretion (Lopez et al., 2008). The tight junctions form an important component of the epithelial monolayer and are a part of the apical junctional complex. They exhibit critical functions like selective solute transport, establishing epithelial polarity and maintenance of different compositions across the apical and basolateral membranes (Rizzolo, 2007; Rizzolo et al., 2011). Our findings of improved morphology, increased PEDF gene expression and presence of tight junctions by Day 5 further support the finding that L‐DOPA, apart from inducing melanosome biogenesis, contributes to improved epithelial cell function.

The advantage of our study is that L‐DOPA is already in clinical use and repurposing of this drug for improving pigmentation of the RPE in albinism patients could help develop a drug‐based therapy to improve vision. Other studies where molecules like pyruvate (Ahmado et al., 2011) and silk sericin (Eidet et al., 2016) have been used still require extensive clinical work to get approval. Moreover, the quality of pigmentation is better in our study. The limitations of the current study are the high concentration of L‐DOPA (1 mM) which also showed some level of nuclear and mitochondrial DNA damage. However, after 5 days post‐pigmentation, the cells continued to remain pigmented with and without L‐DOPA until 28 days (Figure 2c(vii–x)) suggesting the long‐term pigmentation capability of our approach. Such drug reversal experiments have not been performed in other studies to show the long‐term effect and behaviour of de novo induced pigmentation in amelanotic RPE cells. This warrants further study with low concentrations of L‐DOPA for a long period of time to achieve pigmentation. Another limitation of our study is the lack of functional validation confirming that the observed pigmentation is exclusively mediated by tyrosinase activity; future experiments using tyrosinase inhibitors such as kojic acid or cycloheximide will be essential to distinguish enzymatic melanogenesis from L‐DOPA autooxidation.

## 5 CONCLUSIONS

Our study shows that pigmentation can be induced in a model of amelanotic ARPE‐19 cells. This model system can be used to study melanosome biogenesis and melanin synthesis in RPE cells. Using L‐DOPA as a clinically approved drug, pigmentation could be achieved as early as Day 3 of treatment, and it improved by Day 5 of treatment. L‐DOPA could also induce a mature RPE phenotype with improved hexagonal morphology. Application of L‐DOPA in higher concentrations appeared to be cytotoxic, which could be an in vitro or in vivo limitation of our study. Strategies to overcome free radical generation could be used as an alternative mode to decrease the cytotoxic effect observed in our study. Finally, we emphasize the role of L‐DOPA and its potential use to either induce melanogenesis in non‐pigment RPE cells or to improve the function of terminally differentiated RPEs for future therapeutic purpose.

## AUTHOR CONTRIBUTIONS

SG and GP conceptualized the research ideas. SG conducted experiments and prepared the manuscript. GF conducted experiments. SG and GP contributed to the research design creation, literature search and interpretation of the obtained results. All authors read and approved the final manuscript.

## FUNDING INFORMATION

The study was supported by funding from the European Union's Horizon 2020 research and innovation programme under the Marie Skłodowska‐Curie grant agreement and funding allocated to an international project of The Czech Science Foundation (Project Number 18‐04393S), the Norway Grants and Technology Agency of the Czech Republic (KAPPA project TO01000107), the Johannes Amos Comenius Programme (Research Excellence in Regenerative medicine, CZ.02.01.01/00/22_008/0004562) and IAPG institutional support RVO 67985904.

## CONFLICT OF INTEREST STATEMENT

The authors declare that the research was conducted in the absence of any commercial or financial relationships that could be construed as a potential conflict of interest.

## ETHICS STATEMENT

Primary RPE isolation from deceased human donors was approved by the Oslo University Hospital and was performed according to the Helsinki Ethical Protocol. Consent was taken from the kin of the deceased donor for conducting research.

## Data Availability

The raw data supporting the conclusions of this article will be made available by the authors, without undue reservation. The original contributions presented in the study are included in the article. Further inquiries can be directed to the corresponding author.
